# Antitumor activity of chLpMab‐2, a human–mouse chimeric cancer‐specific antihuman podoplanin antibody, via antibody‐dependent cellular cytotoxicity

**DOI:** 10.1002/cam4.1049

**Published:** 2017-03-23

**Authors:** Mika K. Kaneko, Shinji Yamada, Takuro Nakamura, Shinji Abe, Yasuhiko Nishioka, Akiko Kunita, Masashi Fukayama, Yuki Fujii, Satoshi Ogasawara, Yukinari Kato

**Affiliations:** ^1^ Department of Regional Innovation, Tohoku University Graduate School of Medicine2‐1 Seiryo‐machi, Aoba‐kuSendaiMiyagi980‐8575Japan; ^2^ Department of Clinical Pharmacy Practice PedagogyGraduate School of Biomedical SciencesTokushima University1‐78‐1 Sho‐machiTokushima770‐8505Japan; ^3^ Department of Respiratory Medicine and RheumatologyGraduate School of Biomedical SciencesTokushima University3‐18‐15 Kuramoto‐choTokushima770‐8503Japan; ^4^ Department of PathologyGraduate School of Medicinethe University of Tokyo7‐3‐1 HongoBunkyo‐kuTokyo113‐0033Japan; ^5^ Department of ChemistryGraduate School of ScienceChiba University1‐33 Yayoi‐cho, InageChiba263‐8522Japan; ^6^ Department of Antibody Drug DevelopmentTohoku University Graduate School of Medicine2‐1 Seiryo‐machi, Aoba‐ku, SendaiMiyagi980‐8575Japan; ^7^ Project of Antibody Drug DevelopmentNew Industry Creation Hatchery Center2‐1 Seiryo‐machi, Aoba‐ku, SendaiMiyagi980‐8575Japan

**Keywords:** Antibody‐dependent cellular cytotoxicity, chimeric antibody, human podoplanin, monoclonal antibody

## Abstract

Human podoplanin (hPDPN), a platelet aggregation‐inducing transmembrane glycoprotein, is expressed in different types of tumors, and it binds to C‐type lectin‐like receptor 2 (CLEC‐2). The overexpression of hPDPN is involved in invasion and metastasis. Anti‐hPDPN monoclonal antibodies (mAbs) such as NZ‐1 have shown antitumor and antimetastatic activities by binding to the platelet aggregation‐stimulating (PLAG) domain of hPDPN. Recently, we developed a novel mouse anti‐hPDPN mAb, LpMab‐2, using the cancer‐specific mAb (CasMab) technology. In this study we developed chLpMab‐2, a human–mouse chimeric anti‐hPDPN antibody, derived from LpMab‐2. chLpMab‐2 was produced using fucosyltransferase 8‐knockout (KO) Chinese hamster ovary (CHO)‐S cell lines. By flow cytometry, chLpMab‐2 reacted with hPDPN‐expressing cancer cell lines including glioblastomas, mesotheliomas, and lung cancers. However, it showed low reaction with normal cell lines such as lymphatic endothelial and renal epithelial cells. Moreover, chLpMab‐2 exhibited high antibody‐dependent cellular cytotoxicity (ADCC) against PDPN‐expressing cells, despite its low complement‐dependent cytotoxicity. Furthermore, treatment with chLpMab‐2 abolished tumor growth in xenograft models of CHO/hPDPN, indicating that chLpMab‐2 suppressed tumor development via ADCC. In conclusion, chLpMab‐2 could be useful as a novel antibody‐based therapy against hPDPN‐expressing tumors.

## Introduction

Human podoplanin (hPDPN), also known as hT1*α*, hAggrus, or gp36, is expressed in many types of cancers, such as malignant brain tumors, malignant mesotheliomas, lung cancers, esophageal cancers, testicular tumors, bladder cancers, osteosarcomas, and fibrosarcomas 1, 2, 3, 4, 5, 6, 7, 8, 9, 10, 11, 12, 13, 14, 15. hPDPN expression is observed only in squamous cell carcinomas of lung and esophageal cancers, indicating that hPDPN exhibits histological type‐specific expression. The expression of hPDPN in cancer‐associated fibroblasts contributes to a poor prognosis 16, 17, 18, 19, 20, 21. C‐type lectin‐like receptor 2 (CLEC‐2) is an endogenous receptor of hPDPN 22, 23. CLEC‐2 binds to hPDPN through residues Glu47 and Asp48 within its platelet aggregation‐stimulating (PLAG) domain and to *α*‐2,6‐linked sialic acid, which is attached to Thr52 24.

Membrane proteins could be targeted by antibody‐based therapy if (1) they possess cancer‐specific mutations outside the membrane 25, (2) they are overexpressed in cancers rather than in normal tissues 26, 27, 28, or (3) they are posttranslationally modified by phosphorylation or glycosylation 29, 30. Although many anti‐hPDPN monoclonal antibodies (mAbs) are commercially available, most of them react with the N‐terminus of hPDPN, and they do not fulfill the above‐mentioned criteria 6, 31, 32, 33, 34, 35. hPDPN is highly expressed in normal lymphatic endothelial cells (LECs) and normal lung type‐I alveolar cells at the same level as in cancer cells. We previously produced a rat anti‐hPDPN mAb (NZ‐1), which detects hPDPN with high specificity and sensitivity 6, 10, 31. NZ‐1 is efficiently internalized by glioma cell lines and accumulates in tumors in vivo; therefore, it has been suggested to be a suitable candidate for therapy against malignant gliomas 5, 10. Moreover, NZ‐1 inhibits tumor cell‐induced platelet aggregation and tumor metastasis 23. NZ‐1 mediates antibody‐dependent cellular cytotoxicity (ADCC) and complement‐dependent cytotoxicity (CDC) against tumor cells that express hPDPN 36. Furthermore, human–rat chimeric antibodies, such as NZ‐8 and NZ‐12, exhibit high ADCC and CDC in vitro, and they show very high antitumor activities and neutralizing capabilities 36, 37. Nevertheless, these chimeric mAbs are not cancer‐specific; therefore, cancer‐specific anti‐hPDPN chimeric or humanized antibodies should be developed to prevent unfavorable side effects.

We have developed the original technology to produce cancer‐specific mAbs (CasMabs) against hPDPN 29, 38, 39, 40, 41, 42, 43, 44, 45, 46. The established LpMab‐2 recognized the aberrant *O*‐glycosylation and Thr55–Leu64 peptide of hPDPN 29. LpMab‐2 reacted with hPDPN‐expressing cancer cells but not with normal cells, as shown by flow cytometry and immunohistochemistry. Therefore, LpMab‐2 is an anti‐hPDPN CasMab that can be used for molecular‐targeted therapy against hPDPN‐expressing cancers. In immunohistochemistry, less than 10% of hPDPN‐expressing cancer cells were recognized by LpMab‐2. In contrast, another anti‐hPDPN mAb LpMab‐7 reacted with both hPDPN‐expressing cancer cells and normal cells 42. We identified the minimum epitope of LpMab‐7 as Arg79–Leu83 of hPDPN using ELISA, Western‐blot, and flow cytometry. We further produced a human–mouse chimeric anti‐hPDPN mAb, chLpMab‐7 40. chLpMab‐7 showed ADCC and CDC, and inhibited the growth of hPDPN‐expressing tumors in vivo.

In this study, we developed and characterized chLpMab‐2, a human–mouse chimeric anti‐hPDPN antibody derived from LpMab‐2.

## Materials and Methods

### Antibodies

LpMab‐2, a mouse anti‐hPDPN mAb (IgG_1_, kappa), was developed as previously described 29. Human IgG was purchased from Beckman Coulter, Inc. (Fullerton, CA). To generate human–mouse chimeric anti‐hPDPN (chLpMab‐2), appropriate V_H_ and V_L_ cDNAs of mouse LpMab‐2 and C_H_ and C_L_ of human IgG_1_ were subcloned into pCAG‐Ble and pCAG‐Neo vectors (Wako Pure Chemical Industries, Ltd., Osaka, Japan), respectively. CHO‐S/fucosyltransferase 8 (FUT8)‐KO (PDIS‐5) cell lines were generated by transfecting CRISPR/Cas9 plasmids that targeted FUT8 (Target ID: HS0000547010; Sigma‐Aldrich Corp., St. Louis, MO) into CHO‐S cells (Thermo Fisher Scientific Inc., Waltham, MA) using a Gene Pulser Xcell electroporation system. PDIS‐5 cells were screened using *Aleuria aurantia* lectin. Antibody expression vectors were transfected into PDIS‐5 using the Lipofectamine LTX reagent (Thermo Fisher Scientific Inc.). Stable transfectants of PDIS‐5/chLpMab‐2 were selected by cultivating the transfectants in a medium containing 0.5 mg/mL of both geneticin and zeocin (InvivoGen, San Diego, CA). PDIS‐5/chLpMab‐2 cells were cultivated in CHO‐S‐SFM II medium (Thermo Fisher Scientific Inc.). chLpMab‐2 was purified using Protein G‐Sepharose (GE healthcare Bio‐Sciences, Pittsburgh, PA).

### Cell lines

The cell lines LN229, HEK‐293T, NCI‐H226, Met‐5A, Chinese hamster ovary (CHO)‐K1, and P3U1 were obtained from the American Type Culture Collection (Manassas, VA). The LN319 cell line was provided by Prof. Kazuhiko Mishima (Saitama Medical University, Saitama, Japan) 47. Human LECs and PC‐ 10 cells were purchased from Cambrex Corp. (Walkersville, MD) and Immuno‐ Biological Laboratories Co., Ltd. (Gunma, Japan), respectively. LN229 and CHO‐K1 cells were transfected with hPDPN plasmids using Lipofectamine 2000 (Thermo Fisher Scientific Inc.) according to the manufacturer's instructions 29. The LN319/hPDPN‐KO cell line (PDIS‐6) was generated by transfection using CRISPR/Cas9 plasmids (Target ID: HS0000333287) that targeted PDPN (Sigma‐Aldrich, St. Louis, MO), as previously described 48. The cell lines CHO‐S/GnT‐1‐KO (PDIS‐9) and CHO‐S/SLC35A1‐KO (PDIS‐14) were generated by transfecting TALEN and CRISPR/Cas9 plasmids, respectively. The former plasmid‐targeted hsMgat1 (Wako Pure Chemical Industries Ltd.) and the latter targeted SLC35A1 (Target ID: HS0000168432; Sigma‐Aldrich) 49.

The CHO‐K1, CHO/hPDPN, NCI‐H226, PC‐10, and P3U1 cells were cultured in RPMI 1640 medium containing L‐glutamine (Nacalai Tesque, Inc., Kyoto, Japan). The LN229, LN229/hPDPN, LN319, HEK‐293T, and PDIS‐6 cells were cultured at 37°C in a humidified atmosphere containing 5% CO_2_ in Dulbecco's Modified Eagle's Medium containing L‐glutamine (Nacalai Tesque, Inc.) and 10% heat‐inactivated fetal bovine serum (FBS) (Thermo Fisher Scientific Inc.). CHO‐S, PDIS‐9, and PDIS‐14 were cultured in CHO‐S‐SFMII medium (Thermo Fisher Scientific Inc.). LECs were cultured in the endothelial cell medium EGM‐2MV supplemented with 5% FBS (Cambrex Corp.). All media contained 100 U/mL of penicillin, 100 *μ*g/mL of streptomycin, and 25 *μ*g/mL of amphotericin B (Nacalai Tesque, Inc.).

### Flow cytometry

The cell lines were harvested after brief exposure to 0.25% trypsin/1 mmol/L EDTA (Nacalai Tesque, Inc.). After washing with 0.1% BSA in PBS, the cells were treated with primary mAbs for 30 min at 4°C, followed by treatment with FITC‐labeled goat anti‐human IgG (Thermo Fisher Scientific Inc.). Fluorescence data were acquired using Cell Analyzer EC800 (Sony Corp., Tokyo, Japan).

### Preparation of effector cells

Effector cells were prepared as previously described 9. Human peripheral blood mononuclear cells (MNCs) were obtained from leukocytes, which were separated from the peripheral blood of healthy donors. The study with human subjects was approved by the Ethics Committee of Tokushima University.

### ADCC

ADCC was determined with the ^51^Cr release assay 9. Target cells were incubated with 0.1 *μ*Ci of ^51^Cr‐sodium chromate at 37°C for 1 h. After washing three times with RPMI 1640 supplemented with 10% FBS, ^51^Cr‐labeled target cells were seeded in 96‐well plates in triplicate. Human peripheral blood MNCs and chLpMab‐2 or control human IgG were added to the cells. After 6 h of incubation, ^51^Cr released from cells into the supernatant (100 *μ*L) was measured using a gamma counter (PerkinElmer, Waltham, MA). The percentage of cytotoxicity was calculated using the following formula: % specific lysis = (E − S)/(M − S) × 100, where E is the release in the test sample, S is the spontaneous release, and M is the maximum release.

### CDC

CDC was evaluated by the ^51^Cr release assay as previously described 9. The CHO/hPDPN cells (target cells) were incubated with ^51^Cr‐sodium chromate (0.1 *μ*Ci) for 1 h at 37°C. After incubation, the cells were washed with RPMI 1640 supplemented with 10% FBS. The ^51^Cr‐labeled cells were incubated with baby rabbit complement (Cedarlane, Ontario, Canada) at a dilution of 1:32 and chLpMab‐2 (0.01–10 *μ*g/mL) or control human IgG (0.01–10 *μ*g/mL) for 3 h in 96‐well plates. After incubation, ^51^Cr in the supernatant was measured using a gamma counter. The percentage of cytotoxicity was calculated using the following formula: % specific lysis = (E − S)/(M − S) × 100, where E is the release in the test sample, S is the spontaneous release, and M is the maximum release.

### Antitumor activity of anti‐hPDPN antibodies

CHO/hPDPN cells were trypsinized and washed with PBS. The cell density was adjusted with PBS to 5.0 × 10^7^ cells/mL, and 100 *μ*L/animal of the cell suspension was subcutaneously inoculated into BALB/c nude mice. After 1 day, 100 *μ*L of 1 mg/mL of chLpMab‐2 and human IgG were injected into the peritoneal cavity of mice, once a week for 4 weeks (control group, *n* = 6; chLpMab‐2 group, *n* = 6). Human NK cells (5.0 × 10^5^ cells, Takara Bio Inc., Shiga, Japan) were injected around the tumors 4 and 11 days after cell inoculation. The tumor diameter was measured every 3–4 days and was calculated using the following formula: volume = W^2^ × L/2, where W is the short diameter and L is the long diameter. The mice were euthanized 21 days after cell implantation.

### Statistical analysis

All data were expressed as means ± SEMs. Student's *t*‐test, Mann–Whitney U‐test, one‐way ANOVA followed by Tukey–Kramer multiple comparisons, and two‐way ANOVA were performed as appropriate. *P* values less than 0.05 were considered to be statistically significant. All statistical tests were two‐sided.

## Results

### Production of chLpMab‐2

**Figure 1:**
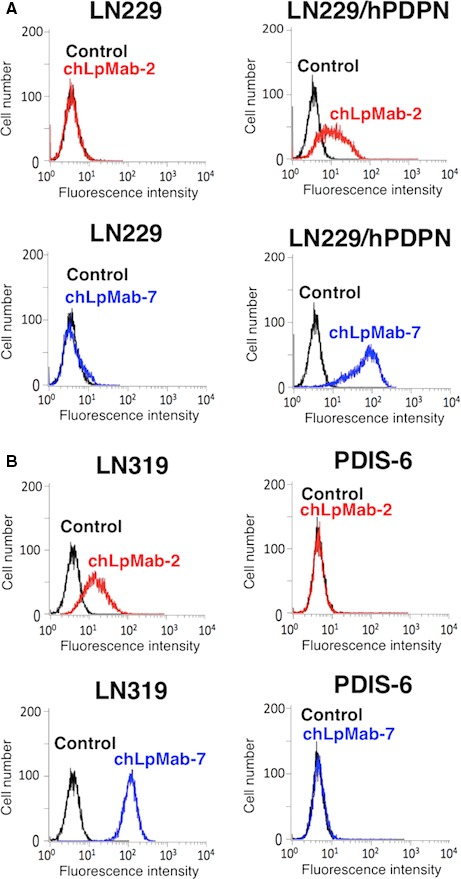
**Flow cytometric analysis using chLpMab‐2 to detect** **hPDPN** **expression**. (A) LN229 and LN229/hPDPN cells were treated with chLpMab‐2 (1 *μ*g/mL, red), chLpMab‐7 (1 *μ*g/mL, blue), and PBS (black) for 30 min at 4°C, followed by treatment with antihuman IgG‐FITC. (B) LN319 and LN319/hPDPN‐KO cells (PDIS‐6) were treated with chLpMab‐2 (1 *μ*g/mL, red), chLpMab‐7 (1 *μ*g/mL, blue), and PBS (black) for 30 min at 4°C, followed by treatment with antihuman IgG‐FITC. Fluorescence data were collected using Cell Analyzer EC800.

We developed chLpMab‐2 from a mouse mAb, LpMab‐2. chLpMab‐2 reacted with LN229/hPDPN cells as revealed by flow cytometry (Fig. 1A). chLpMab‐2 detected endogenous hPDPN in glioblastoma cell line LN319 and not in the LN319/hPDPN‐KO cells (PDIS‐6) (Fig. 1B). Our results showed that chLpMab‐2 was specific against hPDPN.

### Flow cytometric analyses of chLpMab‐2 in cancer and normal cell lines

**Figure 2:**
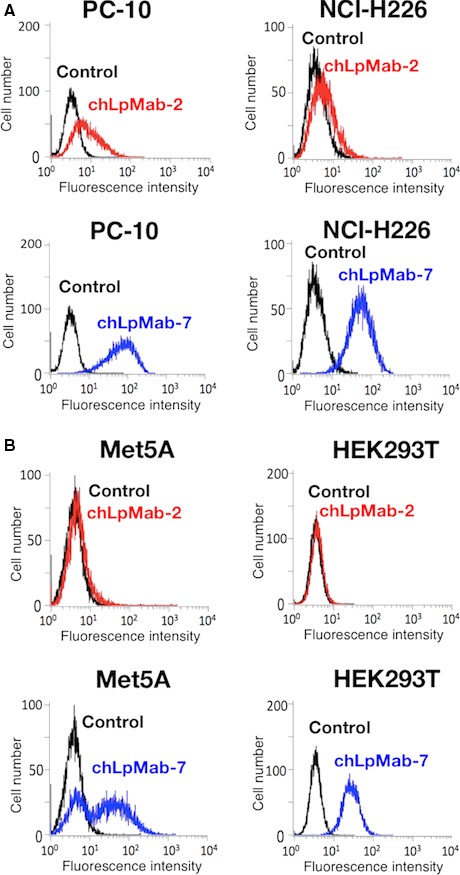
**Flow cytometric analysis using chLpMab‐2 to detect** **PDPN** **expression in human cancer and normal cells**. (A) Human cancer cell lines such as lung squamous cell carcinoma (PC‐10) and mesothelioma (NCI‐H226) were treated with chLpMab‐2 (1 *μ*g/mL, red), chLpMab‐7 (1 *μ*g/mL, blue), and PBS (black) for 30 min at 4°C, followed by treatment with antihuman IgG‐FITC. (B) Normal human cell lines such as mesothelial cells (Met5A) and renal epithelial cells (HEK‐293T) were treated with chLpMab‐2 (1 *μ*g/mL, red), chLpMab‐7 (1 *μ*g/mL, blue), and PBS (black) for 30 min at 4°C, followed by treatment with antihuman IgG‐FITC. Fluorescence data were acquired using Cell Analyzer EC800.

hPDPN is expressed in cancers such as brain tumors and mesotheliomas. By flow cytometry, chLpMab‐2 detected endogenous PDPN in human cancer cell lines such as PC‐10 of lung squamous cell carcinoma and NCI‐H226 of mesothelioma (Fig. 2A). A positive control, chLpMab‐7, detected PDPN expression in two cancer cell lines (Fig. 2A). Because of low hPDPN expression in NCI‐H226 29, the reaction of chLpMab‐2 against NCI‐H226 was much lower than that of chLpMab‐7. In contrast, chLpMab‐2 failed to detect the expression of hPDPN in normal cells such as renal epithelial cells (HEK‐293T) and the mesothelial cell line Met‐5A (Fig. 2B). However, chLpMab‐7 reacted with these cells (Fig. 2B), indicating that chLpMab‐2 was cancer‐specific. These results are consistent with those of our previous study 29.

### Characterization of chLpMab‐2 using glycan‐deficient cell lines

**Figure 3:**
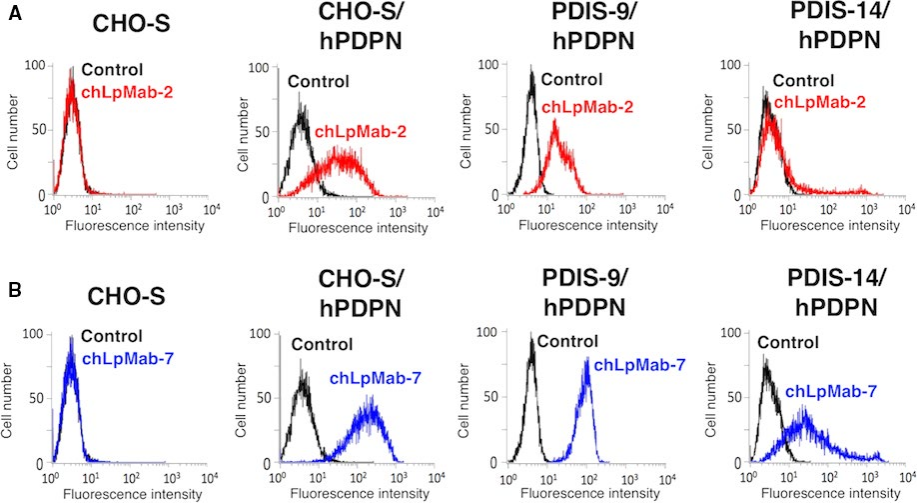
Flow cytometric analysis using chLpMab‐2 to detect hPDPN expression in N‐glycan and sialic acid‐deficient cells. CHO‐S, CHO‐S/hPDPN, PDIS‐9/hPDPN, and PDIS‐14/hPDPN cells were reacted with chLpMab‐2 (A, 1 *μ*g/mL; red), chLpMab‐7 (B, 1 *μ*g/mL; blue), and PBS (black) for 30 min at 4°C, followed by treatment with antihuman IgG‐FITC. Fluorescence data were acquired using Cell Analyzer EC800.

Previous studies have shown that hPDPN is *O*‐glycosylated and not *N*‐glycosylated 6, 24, 50, 51, 52. We used a GnT‐1‐KO cell line (CHO‐S/GnT‐1‐KO, PDIS‐9) and a CMP‐sialic acid transporter (SLC35A1)‐KO cell line (CHO‐S/SLC35A1‐KO, PDIS‐14) 49 to characterize chLpMab‐2. As shown in Figure 3B, chLpMab‐7 reacted with CHO‐S/hPDPN, PDIS‐9/hPDPN, and PDIS‐14/hPDPN cells transfected with the hPDPN expression vector. In contrast, chLpMab‐2 reacted with the CHO‐S/hPDPN and PDIS‐9/hPDPN cells and not with the PDIS‐14/hPDPN cells (Fig. 3A). This result agrees with that of our previous study 29 that the epitope of chLpMab‐2 includes sialic acid.

### ADCC and CDC

**Figure 4:**
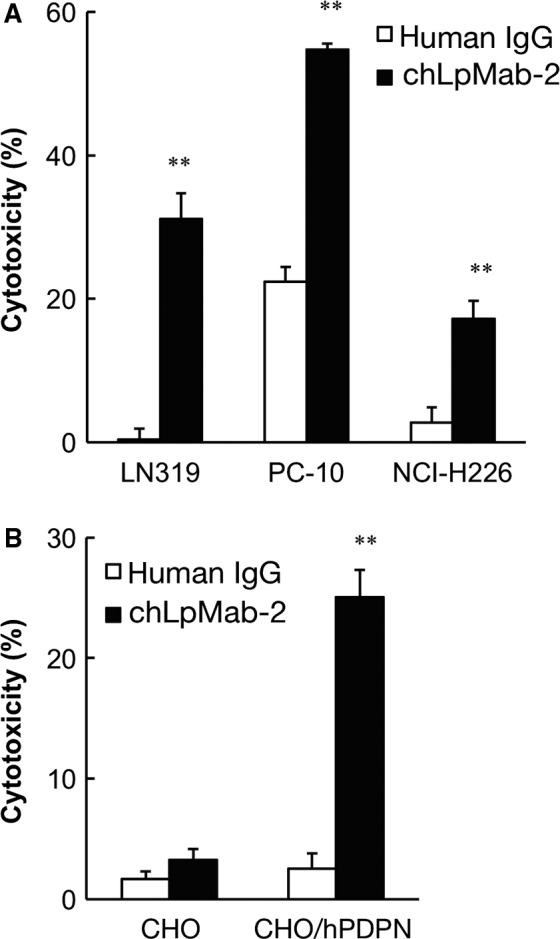
ADCC of chLpMab‐2 against hPDPN‐expressing cell lines. (A) ADCC of chLpMab‐2 using human MNCs against (A) LN319, PC‐10, and NCI‐H226 and (B) CHO and CHO/hPDPN were evaluated by a 6‐h ^51^Cr release assay in the presence of 1 *μ*g/mL of antibody with an E/T ratio of 100. **P <* *0.01

**Figure 5:**
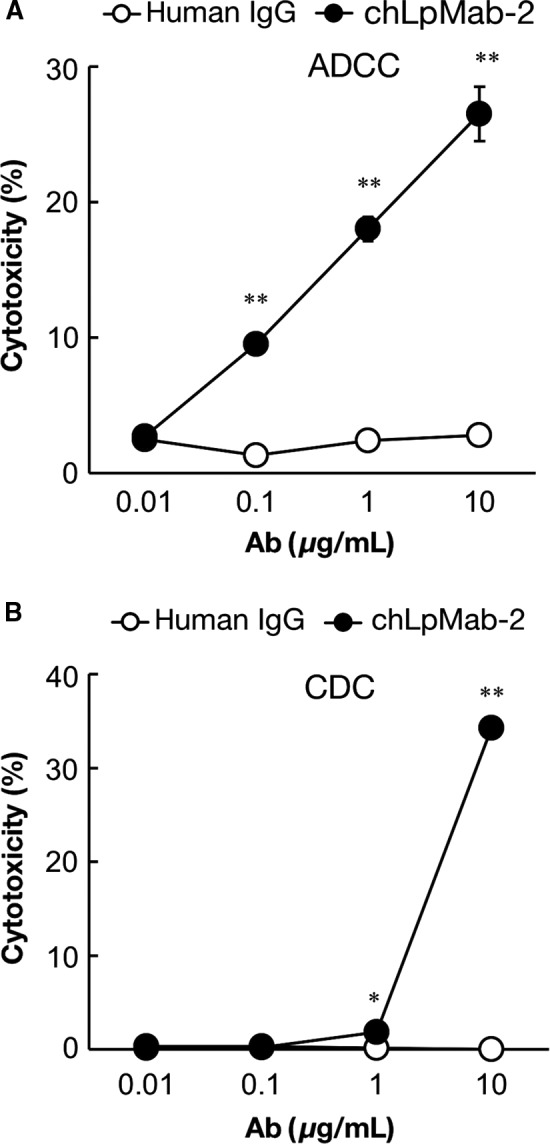
ADCC and complement‐dependent cytotoxicity (CDC) of chLpMab‐2 against CHO/hPDPN cells. (A) ADCC of chLpMab‐2 using human MNCs against CHO/hPDPN was evaluated by a 6‐h ^51^Cr release assay in the presence of 0.01–10 *μ*g/mL of antibody with an E/T ratio of 100. (B) CDC of chLpMab‐2 using baby rabbit complement against CHO/hPDPN was evaluated by ^51^Cr release assay in the presence of 0.01–10 *μ*g/mL of antibody. *P <* *0.05; **P <* *0.01

LpMab‐2 was previously determined to belong to the mouse IgG_1_ subclass that failed to induce ADCC and CDC 29. Therefore, in this study, we converted LpMab‐2 to chLpMab‐2 of human IgG_1_ to study ADCC and CDC 40. As shown in Figure 4A, chLpMab‐2 showed ADCC against the LN319, PC‐10, and NCI‐H226 cell lines. The CHO/hPDPN and CHO‐K1 cells were used to study the specificity of chLpMab‐2 against hPDPN. As shown in Figure 4B, chLpMab‐2 showed ADCC against the CHO/hPDPN cells and not against the hPDPN‐negative CHO‐K1 cells. Furthermore, chLpMab‐2‐induced ADCC against CHO/hPDPN in a dose‐dependent manner (Fig. 5A). In contrast, chLpMab‐2 shows weak CDC against CHO/hPDPN cells (Fig. 5B) and against LN319, PC‐10, and NCI‐H226 cell lines (data not shown).

### Antitumor activity of chLpMab‐2

**Figure 6:**
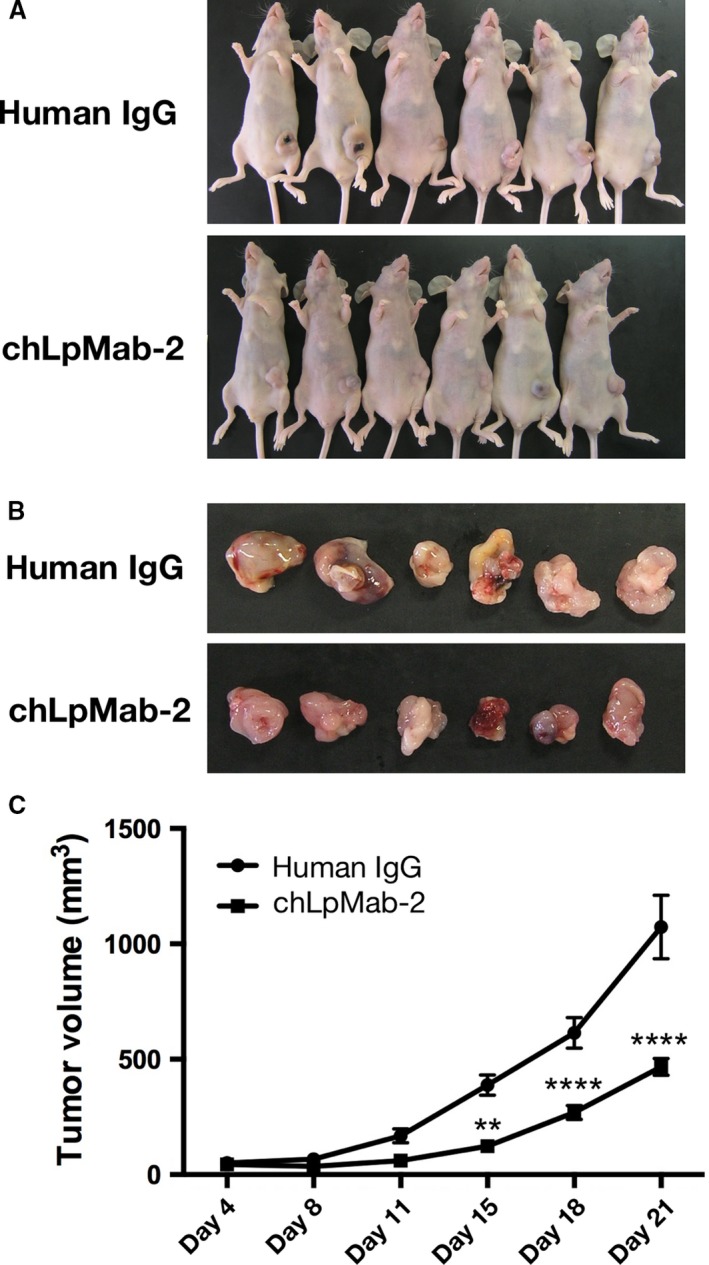
Antitumor effects of chLpMab‐2 on CHO/hPDPN tumor development. CHO/hPDPN cells (3 × 10^6^ cells/100 *μ*L) were subcutaneously inoculated into BALB/c nude mice. After 1 day, 100 *μ*g of chLpMab‐2 or control human IgG was injected into the peritoneal cavity of the mice. The antibodies were injected once weekly for 4 weeks (control group: *n* = 6; chLpMab‐2 group: *n* = 6). The tumor diameter was measured every 3‐4 days and was calculated using the following formula: tumor volume = W^2^ × L/2, where W is the short diameter and L is the long diameter. (A) Comparison of the tumor size and tumor incidence in nude mice (day 21). (B) Comparison of the tumor size (day 21). (C) Primary tumor growth in human IgG and chLpMab‐2‐treated mice. **P <* *0.01; ****P <* *0.001 with two‐way ANOVA.

To study the antitumor activity of chLpMab‐2 on primary tumor growth in vivo, the CHO/hPDPN cells were subcutaneously implanted into the flanks of nude mice. The LN319, PC‐10, and NCI‐H226 cells were not tested because they were not suitable for xenograft models. chLpMab‐2 and control human IgG were injected into the peritoneal cavity of mice, once weekly for 4 weeks (*n* = 6 each), and human NK cells were injected twice around the tumors. Tumor formation was observed in mice from the control and treated groups. However, chLpMab‐2 significantly reduced tumor development compared with control human IgG (Fig. 6A and B). The tumor volume was significantly reduced by chLpMab‐2 treatment on day 15, 18, and 21 (Fig. 6C). These results indicate that the administration of chLpMab‐2 with the NK cells inhibited the primary tumor growth of the CHO/hPDPN cells in vivo.

## Discussion

We previously produced LpMab‐2 (mouse IgG_1_, kappa), one of the CasMabs against hPDPN 29. LpMab‐2 recognized not only the Thr55–Leu64 peptide of hPDPN but also an aberrant *O*‐glycosylated hPDPN, which is attached to Thr55 or Ser56 of hPDPN 48. LpMab‐2 reacted with hPDPN‐expressing cancer cells and not with normal cells, as revealed by flow cytometry and immunohistochemistry 29; therefore, LpMab‐2 is an anti‐hPDPN CasMab that is potentially advantageous for antibody‐based molecular‐targeted therapy against hPDPN‐expressing cancers. However, LpMab‐2 is a mouse IgG_1_; therefore, it cannot be used to study ADCC and CDC against hPDPN‐expressing cancers.

To our knowledge, herein we developed the first cancer‐specific human–mouse chimeric anti‐hPDPN antibody (chLpMab‐2) from LpMab‐2. Previously, we had developed a human–mouse chimeric anti‐hPDPN antibody (chLpMab‐7) 40 and human–rat chimeric anti‐hPDPN antibodies such as NZ‐8 36 and NZ‐12 37. Other groups have reported the development of human–mouse chimeric anti‐hPDPN/hAggrus antibodies that were not cancer‐specific 32, 33. Because hPDPN is expressed in many normal organs such as the lung and kidney, non‐CasMabs against hPDPN are not suitable for antibody‐based molecular targeting therapy.

In this study, we used FUT8‐KO CHO‐S cells (PDIS‐5) to express afucosylated chLpMab‐2. Afucosylated antibodies are known to exhibit high ADCC 53. Consistent with the literature, the ADCC of afucosylated chLpMab‐2 was 3.4 times higher than that of fucosylated chLpMab‐2 (data not shown). In contrast, afucosylated chLpMab‐2 showed a lower CDC (Fig. 5B) than fucosylated chLpMab‐2 (data not shown). Gasdaska et al. have reported that the higher ADCC of afucosylated rituximab suggests an improvement in effectiveness and potency, however, its lower CDC may mitigate infusion toxicity 54. They concluded that afucosylated rituximab was clinically better than fucosylated rituximab. In contrast, Niwa et al. have reported that fucose depletion can provide a panel of IgGs (IgG_1_, IgG_2_, IgG_3_, and IgG_4_) with enhanced ADCC but that none of the IgGs affected CDC 53.

In our previous study, a human–mouse chimeric anti‐hPDPN mAb showed CDC for antitumor activity in the absence of human NK cells in a mouse xenograft model 36. In this study, chLpMab‐2 exhibited ADCC for antitumor activity in the xenograft model with added NK cells 9. Our results indicate that ADCC is important to induce the antitumor activity of anti‐hPDPN CasMab in the xenograft model for two reasons: i) fucosylated chLpMab‐2, which was expressed in CHO‐K1 cells, showed high CDC but failed to induce enough ADCC and antitumor activity against hPDPN‐expressing tumors (data not shown) and ii) afucosylated chLpMab‐2 did not show enough CDC in vitro (Fig. 5B) but exhibited higher ADCC in vitro (Fig. 5A) and antitumor activity against hPDPN‐expressing tumors (Fig. 6).

Taken together, chLpMab‐2 may be useful as a novel antibody‐based therapy against hPDPN‐expressing tumors with no unexpected side effects.

## Conflict of Interest

None declared.
